# RUNX1T1 function in cell fate

**DOI:** 10.1186/s13287-022-03074-w

**Published:** 2022-07-28

**Authors:** Nan Hu, Linqing Zou, Cheng Wang, Guoqi Song

**Affiliations:** 1 Department of Hematology, Affiliated Hospital and Medical School of Nantong University, Nantong, 226001 Jiangsu China; 2 Department of Human Anatomy, Jiangsu Key Laboratory of Neuroregeneration, Nantong University, Nantong, 226001 Jiangsu China

**Keywords:** RUNX1T1, Cell fate, Progenitor cells, Development, Differentiation

## Abstract

RUNX1T1 (Runt-related transcription factor 1, translocated to 1), a myeloid translocation gene (MTG) family member, is usually investigated as part of the fusion protein RUNX1-RUNX1T1 for its role in acute myeloid leukemia. In the main, by recruiting histone deacetylases, RUNX1T1 negatively influences transcription, enabling it to regulate the proliferation and differentiation of hematopoietic progenitors. Moreover, the formation of blood vessels, neuronal differentiation, microglial activation following injury, and intestinal development all relate closely to the expression of RUNX1T1. Furthermore, through alternative splicing of *RUNX1T1*, short and long isoforms have been noted to mediate adipogenesis by balancing the differentiation and proliferation of adipocytes. In addition, RUNX1T1 plays wide-ranging and diverse roles in carcinoma as a biomarker, suppressor, or positive regulator of carcinogenesis, closely correlated to specific organs and dominant signaling pathways. The aim of this work was to investigate the structure of RUNX1T1, which contains four conserved nervy homolog domains, and to demonstrate crosstalk with the Notch signaling pathway. Moreover, we endeavored to illustrate the effects of RUNX1T1 on cell fate from multiple aspects, including its influence on hematopoiesis, neuronal differentiation, microglial activation, intestinal development, adipogenesis, angiogenesis, and carcinogenesis.

## Introduction

*RUNX1T1*, also known as *ETO, MTG8,* or *CBFA2T1*, is a member of MTG family [1]. Homologs of the MTG family include *RUNX1T1 (MTG8)*, *MTG16* (*ETO2*), characterized by homologous sequences of *MTG8* on chromosome 16 and *MTGR1* (also *ETOR1/EHT/CBFA2T2*), a paralog of *MTG8* [2]. MTG family members share four conserved structural domains (NHR1–4) and combine with a similar set of factors, including DNA-binding transcription factors, histone deacetylases (HDACs), and corepressor complexes, but their biological roles and expression patterns are distinct from each other [3]. This article focuses mainly on what is known about RUNX1T1.

*RUNX1T1* mRNA is expressed in many normal tissues, especially brain, heart, skeletal muscle, and adipose tissue, with the brain and heart exhibiting the highest expression level [4]. Although first noted for its role in neurogenesis, *RUNX1T1*, in the form of *RUNX1-RUNX1T1*, has been recently widely researched in association with hematopoiesis and acute myeloid leukemia (AML) [5, 6]. RUNX1T1 is expressed lowly in normal hematopoietic cells, while in patients with AML, *RUNX1-RUNX1T1*, is highly expressed. This fusion gene is present in 4%–12% of adult and 12%–30% of pediatric patients according to reported cases of AML [7].

The effects of *RUNX1T1* on hematopoiesis are associated not only with the reduced function of *RUNX1* but also *RUNX1T1* itself. Although *RUNX1T1* does not interact directly with DNA, it can be recruited by transcription factors, including growth factor independence-1 (GFI1), a critical erythroid transcription factor, and B-cell lymphoma-6 (BCL6), promyelocytic leukemia zinc finger protein (PLZF), and form multi-protein compound [1]. In turn, *RUNX1T1* recruits HDACs as assistant transcriptional genes [8], which negatively influence gene transcription by changing the chromosome structure and inhibiting the binding of transcription factors with DNA [7]. By mediating HDACs and DNA methyltransferase-1 (DNMT1), *RUNX1T1* regulates histone deacetylation and methylation of DNA histone, leading to transcription silencing, the modulation of which enables *RUNX1T1* to have various effects in different situations [7]. In this article, based on its structure, the functions of *RUNX1T1* in cell fate will be illustrated over hematopoiesis, nervous system, intestinal development, adipose metabolism, and carcinoma.

## RUNX1T1 gene and protein structure

**Fig. 1:**
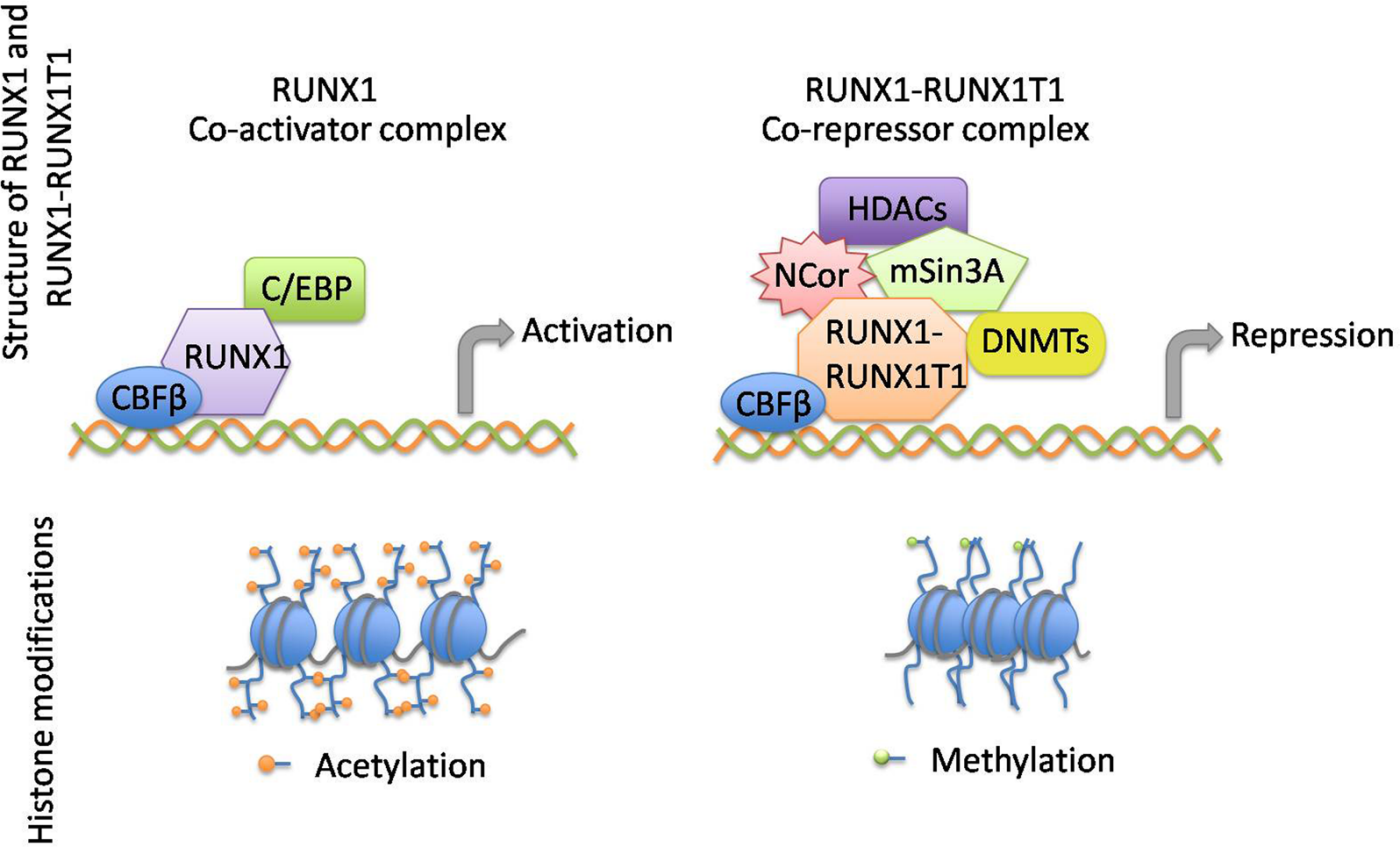
The structure and transcription factor complexes of RUNX1 and RUNX1-RUNX1T1 along with histone modifications at different states. Although RUNX1T1 does not bind directly with DNA, it assists the combination of RUNX1-RUNX1T1 with N-CoR and DNMTs to further recruit HDACs and transform from a co-activator to co-repressor complex. When the CRD binds with m-Sin3A and recruits HDACs, the histone is methylated and binds closer to DNA, tightening the chromosomal structure and leading to transcriptional silence

*RUNX1T1* is located in 8q22 and about 136 kb in length. It contains zinc structures, transcription-activated domains that are rich in proline, and 13 exons that extend to about 87 kb [9]. The structure of RUNX1T1 comprises four conserved nervy homolog domains, namely Nervy homology regions 1–4 (NHR1-4), derived from the *Drosophila melanogaster* genome [8]. NHR1, homologous to the TATA box-related gene TAF110 and other TAF genes of *D. melanogaster*, is located close to the N-terminals of the domains and acts by combining with E-protein [10]. NHR2, known as a hydrophobic heptad repeat (HHR), functions by assisting members of the MTG family to form homologous or heterologous dimers. Together with nearby sequences at the N- and C-terminals (236–432aa), NHR2 is regarded as a core repressor domain (CRD) [11]. This region has clear inhibitory ability and interacts strongly with mSin3a, which enables N-CoR/SMRT to recruit HDACs, deacetylating the chromatin histone of the target gene promoter or enhancer [12]. NHR3, containing a putative amphipathic helix, features in the interaction of A-kinase anchor proteins with RIIα and functions by partially binding to co-repressors [13]. The NHR4 domain, also regarded as a myeloid-Nervy-DEAF1 homology domain (MYND), exhibits two zinc structures at the C terminal, which assist in the combination of RUNX1T1 with the nuclear core repressor (N-CoR) and DNMTs to further recruit HDACs and form a co-repressive compound [14]. After the binding of the CRD with mSin3A and effective recruitment of HDACs, histone is methylated to induce a closer bind with DNA, which tightens the spatial structure of the chromosome and makes it difficult for transcription to be triggered. Through translocation t(8;21)(q22;q22), the N-terminal of 21q22 attaches to chromosome 8, with the C-terminal containing nearly a full length *RUNX1T1*, while another derivative chromosome 21 with 8q22 translated being transcriptionally inactive [6]. The translocation combines nearly the entire RUNX1T1 open reading frame and DNA-binding Runt domain of RUNX1, including its binding sites with CBFβ and CCAAT enhancer-binding protein(C/EBP), which deprives RUNX1 of part of its binding domains and replaces the co-activator complex of RUNX1 with the RUNX1T1 co-repressor complex (Fig. 1) [8, 12].

Moreover, crosstalk between RUNX1T1 and other signaling pathways enables the transfer of information from outside cells into cells, further regulating the process of self-renewal or differentiation [10]. In most populations of post-mitotic cells, RUNX1T1 is expressed in a constant pattern and involved in the inhibition of basic helix-loop-helix gene transcription [1]. During the activation of Notch, the RBP-J complex, a crucial mediator of RUNX1T1 interaction with the Notch signaling pathway, switches Notch from silenced to activated state. Next, Mastermind binds to the Notch/RBP-J compound and collaborates with it to further recruit activators, including mediators, chromatin remodeling factors, and histone transferase [15, 16]. Although RUNX1T1 is regarded as a member of the RBP-J corepressor complex, which is endogenously DNA bound, the direct connection between RUNX1T1 and RBP-J remains unknown. It is proposed that this interaction might depend on the protein SHARP (SMRT- and HDAC1-associated repressor protein), which has been demonstrated to interact with both RUNX1T1 and RUNX1-RUNX1T1 [16, 17]. Thus, as a platform for various types of transcription repressors, RBP-J/SHARP may bind with RUNX1T1, localize to the promoter domain of Notch, and then negatively affect Notch target genes. However, RUNX1-RUNX1T1 may reverse this negative function, and further upregulate certain Notch target genes like *Hes1, Hes5*, *or Hey1* [17, 18]. Detailed functions of RUNX1T1 under physiological or pathological conditions will be illustrated below.

## The effects of RUNX1T1 on hematopoiesis

The effects that RUNX1T1 exerts on hematopoiesis are complicated, including modulation of proliferation and differentiation of hematopoietic progenitor cells (HPCs) mainly in its fusion form. Taking up 12% of all cases, the translocation t (8; 21)(q22; q22) is a commonly existing chromosomal abnormality found in the fusion oncogene of RUNX1-RUNX1T1, intimately relating to the AML M2 subtype in the FAB classification, which is feathered by granulocytic maturation (Gr-1 ≥ 30%) in differentiation property and morphology [19]. In murine and zebrafish models, RUNX1-RUNX1T1 is found to cause increased proliferation of HPCs, growth arrest of myeloid and granulocytic differentiation, leading to a clonal expansion of immature myeloid cells and accumulation of immature granulocytes [20, 21]. RUNX1-RUNX1T1 degradation would trigger acceleration of myeloid differentiation and carcinogenesis by suppressing the normal cell cycle, pushing instead for the self-renewal of HPCs [22, 23]. Furthermore, the expression of RUNX1-RUNX1T1 mRNA on a single leukemia cell during the clinical onset period has also been shown to be notably highly than that under remission state [24]. In recent studies, flow cytometry has been utilized to examine the efficiency of inducing RUNX1-RUNX1T1 by injecting tamoxifen (TMX, 0.05 mg/g), along with the surrogate marker GFP. Through the data analysis of white blood cell count and GFP expression in the peripheral blood of TMX-induced mice, the amount of RUNX1-RUNX1T1 positive cells experienced age-dependent upregulation with senior mice cohort presenting comparably incomplete penetrance and longer latency [19]. The fusion form of the gene deprives RUNX1 of its normal function as the master regulator of hematopoiesis both in primitive and definitive stages, regulating proper specification of hematopoiesis lineages during embryogenesis [20]. Along with reduced expression of the erythroid marker TER119, absence of RUNX1 disturbs the formation of original hematopoietic lineages, resulting in absent macrophages, reduced megakaryocytes, abnormal expression of erythrocytes, and interruption of the hematopoietic program, which transits hematopoietic endothelium to HPCs [25].

**Fig. 2:**
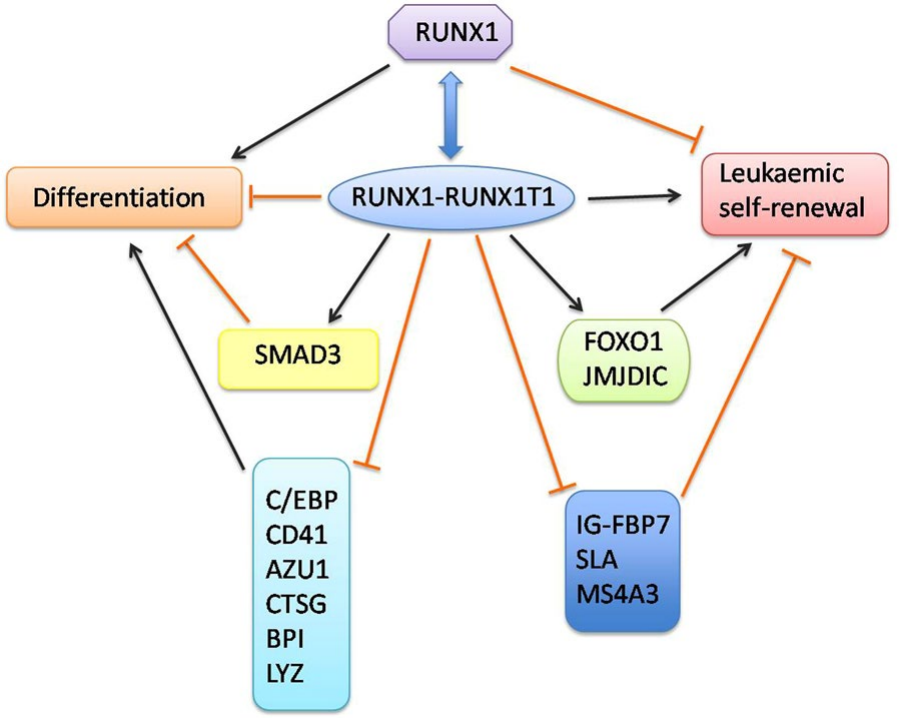
Dynamic interaction among mediators of the RUNX1 and RUNX1-RUNX1T1 network. When interfering RUNX1-RUNX1T1 binds with siRNAs, genes that correspond to granulocytic differentiation or anti-renewal undergo upregulation. However, factors including FOXO1, JMJD1C, and SMAD3 are significantly activated to drive leukemic self-renewal or block myeloid differentiation

The core mediators of the RUNX1-RUNX1T1 transcriptional network comprise nearly 60 genes, including those that regulate monocytic and erythrocytic lineage differentiation and decide cell fate [23]. By downregulating SPI1, PU.1, GATA1, and CEBPA, as well as cytokine-induced upregulation of M-CSF and CD11b receptors, RUNX1-RUNX1T1 disturbs normal hematopoiesis and blocks myeloid differentiation [22, 23]. However, factors such as FOXO1 (forkhead box protein 01) and JMJD1C, a type of histone demethylase, are endogenously activated to drive self-renewal of leukemia stem cells [26, 27]. The cell cycle, from the G1 to S phases, mediated by cyclin-dependent kinases 2 (CDK2), is interrupted by the RUNX1-RUNX1T1-induced upregulation of CDKN1A (p21) [6, 28]. When the fusion protein binds with SMAD3, TGF/vitamin3-dependent myeloid differentiation is blocked [29]. C/EBP functions in the development of granulocytes and is highly expressed in bone marrow. By combining with C/EBP, *RUNX1-RUNX1T1* leads to its suppression as well as that of downstream genes [7, 22]. The upregulation of differentiation-related genes is closely related to decreased binding to *RUNX1-RUNX1T1* and increased binding to C/EBP [7]. When *RUNX1-RUNX1T1* binding domains are interrupted by small-interfereing RNAs (siRNAs), genes relevant to granulocytic differentiation, including *AZU1, CTSG, BPI, RNASE2, LYZ*, or anti-proliferation, such as *IG-FBP7, SLA*, *and MS4A3*, experience upregulation [30]. In addition, depletion of *RUNX1-RUNX1T1* results in increased susceptibility to myeloid differentiation and upregulated expression of C/EBP and cytokines like CD41 and the p14 promoter (Fig. 2) [6, 20]. With RUNX1-RUNX1T1 expression, the expected block of differentiation has appeared on CD34 + human cord blood progenitor cells while coming into the differentiation state, with the fusion gene being degraded by CRISPR-Cas9 technology, which may provide novel insights into alternative treatment strategies for M2 AML [20, 23].

## The effects of RUNX1T1 on the nervous system

RUNX1T1, which is most highly expressed in the brain and heart, was first noted for its role in neurogenesis [5]. All three members of the MTG family, namely *MTG8, MTG16,* and *MTGR1*, are found to express in different patterns during neurogenesis [8]. Through analyses of *RUNX1T1* expression pattern in chick and Xenopus, MTG family members are expressed in a cascade during neuronal differentiation, with *MTGR1* being initially upregulated during neuronal progenitor transformation into differentiated post-mitotic neurons and downregulated during the definitive stage, while the expression of *RUNX1T1* and *MTG16* increases, indicating their function in post-mitotic neurons [1]. Similarly, in chick, *RUNX1T1* is mainly expressed in differentiated neurons within the mantle zone, corresponding to the position of post-mitotic motor neurons. However, immunohistochemical analysis of the mouse embryo has demonstrated that RUNX1T1 protein exists not only in the nucleus of developing brain and spinal cord neural cells but also in the cytoplasm of cerebellum sections and synaptosomal fractions of the forebrain [31]. Although the mechanism that modulates the subcellular localization is still unclear, it is reasonable to believe that *RUNX1T1*, along with *MTG16* and *MTGR1*, is involved in neurogenesis, especially at the definitive stage.

Through immunofluorescence experiments, *RUNX1T1* has been found to be expressed in all hippocampal radial glial cells (RGCs) and to exist in neurons but not in astrocytes in vitro or in vivo. When imitating hippocampal microenvironment denervation damage in vitro, the expression levels of *RUNX1T1* mRNA and protein on hippocampal RGCs increase significantly, with more hippocampal RGCs differentiating to MAP-2 + neurons, but still failing to reverse the damage [1, 4]. When *RUNX1T1* was utilized to increase *RUNX1T1* expression in hippocampal RGCs in vitro, over 30% cells were shown to differentiate into MAP-2 + neurons, significantly higher than that found in the control group. Correspondingly, when *RUNX1T1* was knocked out, the opposite results appeared [4]. Hence, silenced *RUNX1T1* expression probably downregulates neural differentiation, while its overexpression likely promotes it. Moreover, *RUNX1T1* mutation has been inferred as a possible explanation for cognitive disorder according to the sequencing analysis of genomic hybridization. A subsequent report claimed that a translocation breakpoint within intron 1b or deletion from exons would probably lead to *RUNX1T1* function impairment, causing a mild to moderate level of intellectual disability [32].

Apart from mediating neuronal cellular development, *RUNX1T1* is closely related to microglial cell development, which commonly appears in gray matter and takes up 5%–20% of the central nervous system cell population [33]. During microglial development, *RUNX1T1* has been found mostly to exist in amoeboid microglial cells of rat brains during the postnatal stage, while being difficult to detect in ramified microglia cells of adult brains. The protein was observed to translocate to the nuclei when activated [34]. Meanwhile, *RUNX1* also experienced a progressive loss as microglia morphologically transformed from the amoeboid to ramified form. However, after traumatic nerve injury, *RUNX1* and *RUNX1T1* are both upregulated simultaneously upon microglial cell activation [35]. Recent studies have focused on silencing *RUNX1T1* expression to inhibit its promotion of the inflammatory response following microglial activation. When *RUNX1T1* is knocked out by siRNA, CDK4 and proliferation index expression increases in activated microglia cells [36]. CDK4, which regulates the cell cycle transition of G1/S by binding with cyclin D1 and phosphorylates retinoblastoma protein (pRb), can be upregulated under the effect of HDAC inhibitors [37]. Moreover, the depletion of *RUNX1T1* enhances the expression of L-aminoacid transporter-2 (LAT2), increasing its ability to prevent NO production and protecting microglial cells from neurotoxic effects [36]. Nonetheless, as shown by a chromatin immunoprecipitation (ChIP) assay, when microglial cells are activated, RUNX1T1 increasely binds with LAT2 promoter and restricts LAT2 from preventing NO production. This upregulation of RUNX1T1 binding does not occur under the influence of HDAC inhibition, indicating that RUNX1T1 is involved in microglial activation probably by mediating HDACs and downstream factors [36]. In fact, various types of HDAC inhibitors, such as suberoylanilide hydroxamic acid (SAHA) and its structural analog ITF2357, have proven anti-inflammatory effects, providing a target for treatment from neurotoxic effects caused by microglial activation [38].

## RUNX1T1 and intestinal development

**Fig. 3:**
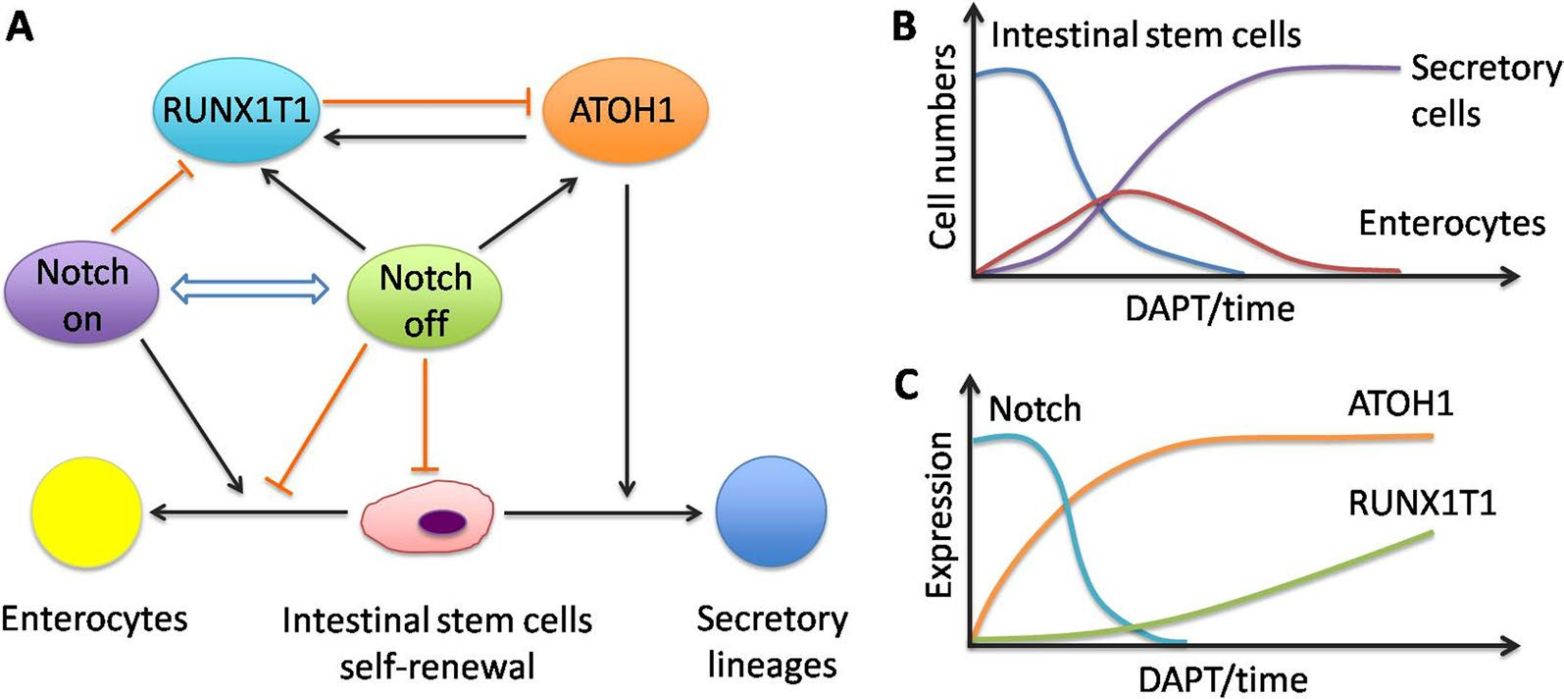
RUNX1T1 regulates the cell fate choice of intestinal progenitor cells in collaboration with Notch and ATOH1 (**A**). The activation of Notch steers differentiation towards enterocytes, while its inactivation represses the self-renewal program of stem cells and de-represses ATOH1 and RUNX1T1, promoting differentiation towards secretory lineages (**B**). When Notch is interfered with by DAPT, ATOH1 is de-repressed and undergoes rapid upregulation, followed by increased RUNX1T1 expression. Meanwhile, the intestinal stem cells tend to differentiate into secretory cells (**C**)

Studies on the intestinal organoids of mice and humans have demonstrated that RUNX1T1 controls the binary fate decision of intestinal progenitor cells—whether to differentiate into enterocyte or secretory lineages. By adding an inactivating insertion of *Lac Z* in exon 2 of the *RUNX1T1* locus, mutant mice suffered from serious defects and deficiencies in most parts of the intestine. In the established *RUNX1T1* knockout mouse model, significant defects in gut structure and rectal hemorrhage appeared, as well as impairment of intestinal stem cells differentiation, which led to a decline of postnatal survival [39]. The fate choice process of progenitor cells is purported to be related to ATOH1, a dominant regulator of the secretory lineages, and Delta-like (Dll), one of the Notch ligands [40]. Notch is activated during intestinal stem cell differentiation, whose activation can direct differentiation into enterocytes. However, when the intestinal organoids are treated with DAPT, a Notch inhibitor, ATOH1 is derepressed and experiences rapid upregulation, followed by increased RUNX1T1 expression. Moreover, similar results appear when Notch is inactivated by other means including deletion of RBP-J, or by utilizing dibenzazepine (DBZ), a γ-secretase inhibitor, indicating that ATOH1 and RUNX1T1 can both be repressed by Notch [17, 18]. The upregulation of RUNX1T1 has been found to occur much later than that of ATOH1, and this tendency disappears when blocking ATOH1, suggesting that ATOH1 might be capable of mediating RUNX1T1 expression [39]. When Notch is inactivated, the upregulation of ATOH1 and RUNX1T1 represses the self-renewal program of stem cells and promotes secretory lineage differentiation (Fig. 3). However, driven by the characteristic pattern of Notch activation, regarded as lateral inhibition, and the upregulation of ATOH1, the surrounding cells reach the opposite cell fate. Therefore, although ATOH1 has the tendency to drive differentiation towards secretory lineage, its neighbors push the cell fate decision towards enterocytes [39]. This process has been proposed to be fulfilled by RUNX1T1 and MTG16, which inhibit ATOH1 expression by occupying its enhancer and many other binding sites like Neurog3 and Gfi1. In this way, when RUNX1T1 and MTG16 become dominant regulators, the fate choice of intestinal progenitor cells is steered towards enterocyte differentiation and maturation, which helps to maintain balance in normal intestinal development. Therefore, by modulating Notch, ATOH1 and RUNX1T1 expression, it might be possible to mediate intestinal progenitor differentiation or maintain their multi-potency as stem cells [39].

## RUNX1T1 and adipogenesis

**Fig. 4:**
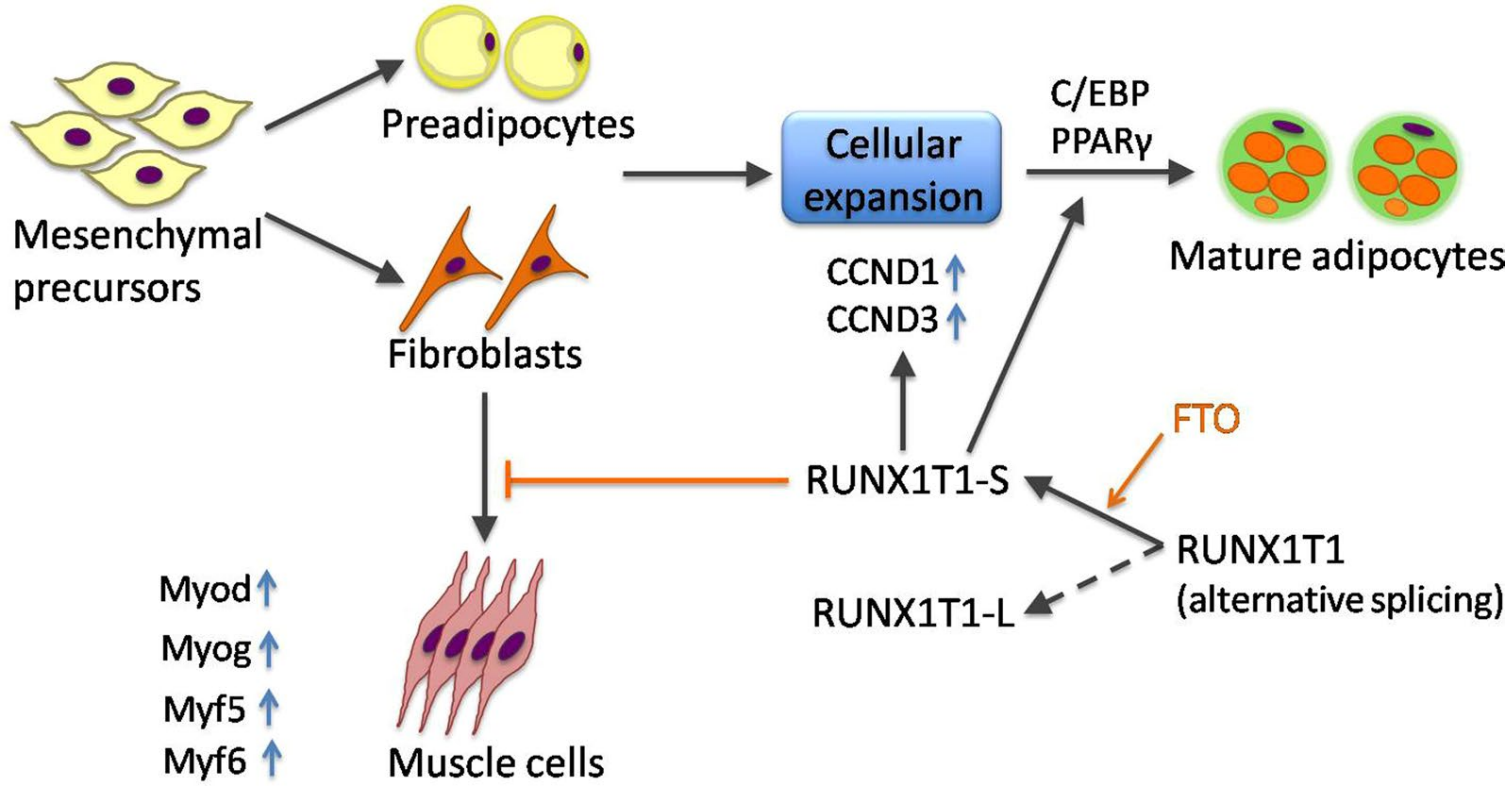
The effects of RUNX1T1 isoforms on adipogenesis via alternative splicing of RUNX1T1 mRNA. Adipogenesis covers the transition from mesenchymal precursors to fibroblasts and preadipocytes and the final differentiation into adipocytes. Overexpression of FTO leads to an increase in the RUNX1T1-S isoform, which promotes the process of cellular expansion by upregulating CCND1 and CCND3 and differentiation into mature adipocytes through interaction between PPARγ and C/EBP, as well as myogenesis inhibition

RUNX1T1 is also involved in maintaining the metabolic balance of various nutrients, among which, adipogenesis is vital and indispensable. Adipogenesis involves the transition of mesenchymal precursors to preadipocytes and the terminal differentiation of preadipocytes. It has been found that RUNX1T1 exerts multiple effects on adipogenesis by initially suppressing the activity of C/EBP and regulating preadipocyte differentiation according to the balance of different *RUNX1T1* isoforms via alternative splicing [41–43]. Normally, preadipocytes experience cellular hypertrophy and differentiate into mature adipocytes postnatally, while their proliferation mainly occurs during late gestation [44]. The differentiation of adipocytes involves interaction between peroxisome proliferator-activated receptor gamma (*PPARγ*) and C/EBP, the inhibition of which either by *RUNX1T1* or siRNAs significantly interrupts normal adipogenesis [42]. Brown adipocytes have been found to originate from a *Myf5*-positive, fibroblastic lineage according to a lineage tracing study. However, the process of adipogenesis is drastically impaired when *miR-193b/365* is blocked, resulting in the induction of skeletal myogenesis as indicated by the upregulation of several myogenesis markers, including *Myod, Myog, Myf5, Myf6,* and *Ckm*, as well as the upregulation of *miR-193b/365* target genes, including *RUNX1T1* [45]. As C/EBP inhibitor, RUNX1T1 interferes with the final differentiation of preadipocytes as well as brown fat adipogenesis. Moreover, RUNX1T1 expression undergoes downregulation upon the transition of pluripotent mesenchymal precursors to preadipocytes [43]. As a mediator of adipogenesis, *RUNX1T1* modulates adipocyte differentiation through its alternative isoforms, with mutual feedback between fat mass and obesity-associated (FTO) genes, which encode nucleic acid demethylase and are capable of mediating *RUNX1T1* mRNA splicing by demethylating N6-methyladenosine [46]. The overexpression of FTO is accompanied by a significant increase in the short (S) isoform of *RUNX1T1*, which accelerates the progression of adipocyte formation, probably via upregulating CCND1 and CCND3; however, no clear changes in the expression of the long (L) isoform has been found in mouse embryonic fibroblasts (Fig. 4). Nevertheless, overexpressing the L isoform impairs the formation of adipocytes [47]. Research on the ovine *RUNX1T1* gene has further illustrated the effects of *RUNX1T1* on adipogenesis. By cloning the ovine *RUNX1T1* gene and obtaining its coding sequence, the S isoform has been observed to be 245 bp shorter than the L isoform. Corresponding to the results from mouse experiments, the L isoform is negatively related to adipogenesis, and its knockdown results in the promotion of lipid accumulation as well as preadipocyte differentiation [41]. Moreover, since expression of the L-isoform is higher than that of the S-isoform, the dominant effect of *RUNX1T1* is more likely to emerge as a negative regulator, which helps to explain why overexpressing *RUNX1T1* was shown to inhibit adipogenesis by attaching to C/EBP at its DNA binding site [43, 46]. Therefore, both the differentiation and proliferation of adipocytes might depend on the balance between the S and L isoforms of *RUNX1T1*, which might be not only essential to the stability of normal metabolism but also potential as a target for foam cell generation and development of lipid disorder diseases like atherosclerosis [48].

## The effects of RUNX1T1 on the formation of blood vessels

RUNX1T1 has been found to be involved in the regulation of two stages of blood vessel formation, vasculogenesis and angiogenesis, during which several factors are required to accelerate the transition, including fibroblast growth factors (FGF), vascular endothelial growth factor (VEGF), and BMP4 [49]. VEGF not only regulates vasculogenesis but also functions as a survival factor for endothelial cells (ECs) [50]. Endothelial progenitor cells (EPCs), which comprise two sub-groups, endothelial colony-forming cells (ECFCs) and circulating angiogenetic cells (CACs), gradually differentiate into ECs during vessel generation or injury recovery. Cultivated from cord blood, ECFCs normally present high levels of *RUNX1T1* expression, while in heterozygous *RUNX1T1* knockout mice, the motility and viability of ECFCs along with the formation of embryonic vasculatures experience downregulation, leading to the failure of vascular recovery following ischemic injury. *RUNX1T1* is involved angiogenetic activities as an essential regulator of motility, viability of ECFCs, vessel permeability, and tube formation [49]. The correlation between *RUNX1T1* and angiogenetic abilities is positive, with *RUNX1T1* promoting the survival and motility of ECs. Knocking out *RUNX1T1* not only results in the impairment of vessel-forming ability, the reduction of tube lengths and branch numbers but also leads to an increase in vessel permeability and a decrease in aorta thickness [50]. Through interference sequence transduction against *RUNX1T1*, angiogenetic factors including BMP-4, VEGFA, TGF-β2, Angiopoietin-2, and HBEGF were shown to experience downregulation in heterozygous *RUNX1T1* knockout mice, indicating that *RUNX1T1* likely mediates the angiogenetic capabilities of ECFCs through activation of these angiogenetic factors or epigenetic regulation via protein–protein interaction [49, 50].

## RUNX1T1 and carcinoma

The roles played by RUNX1T1 in carcinogenesis are diverse and tightly connected to specific organs and signaling pathways. RUNX1T1 has been widely revealed as a regulator or a biomarker during carcinogenesis. Considering the complex signaling pathways that RUNX1T1 is involved in and its various interactions with other molecules, the effects that RUNX1T1 has on carcinogenesis may be associated with the regulation of downstream factors and epigenetic methylation of the *RUNX1T1* gene or histone through recruitment of HDACs to mediate their acetylation or deacetylation state [14, 51–54].

RUNX1T1 is collectively viewed as a biomarker for primary pancreatic endocrine tumors (PETs), breast cancer, and colorectal cancer (CRC) and a strong indicator of patient prognosis [51, 54, 55]. The analyses all connect a higher level of *RUNX1T1* with a comparably positive prognosis, while lower levels tend to indicate a negative prognosis. By modulating the expression of pancreatic polypeptide and ghrelin, RUNX1T1 is expressed lowly in liver-metastatic PETs [51]. Breast cancer patients who are treated with tamoxifen are more likely to have higher levels of *RUNX1T1* expression, with longer distant metastasis-free survival and relapse-free survival, while those suffering from triple-negative *(ER − /PR − /HER2 −)* or estrogen receptor α *(ERα)*-knockdown breast cancer with worse prognosis tend to show lower levels of *RUNX1T1* expression [55]. When it comes to CRC, however, *RUNX1T1* is proven to be a tumor-suppressive gene, the re-expression of which causes a significant decrease in CRC cell growth and proliferation as well as increased sensitivity to 5-flurouracil [54].

In recent studies, *RUNX1T1* has been shown to be more likely to function as a suppressor during the advance of gastric tumors, gliomas, and ovarian cancer. In gastric cancer, the role of RUNX1T1 is related to C/EBPβ, which is overexpressed in AML, gastric, skin and bladder cancer [56, 57]. By combining with cyclin D1, C/EBPβ realizes its oncogenic effect by repressing the differentiation marker and promoting cell proliferation, leading to the switch from stomach epithelial differentiation to proliferation [57]. However, the occurrence of gastric cancer is commonly accompanied by an increased level of cyclooxygenase-2 (COX2) and a decrease in mucous-associated protein trefoil factor 1 (TFF1), a tumor suppressor. When RUNX1T1 combines with C/EBPβ, its binding with DNA and repression of the TFF1 promoter gene are significantly interrupted, which reduces the proliferation of gastric cancer cell lines and slows oncogenesis [58]. However, according to observations of human gastric cancer samples, RUNX1T1 is commonly methylated, probably worsening the situation [57]. In glioma, RUNX1T1 has been viewed as a potential biomarker and possible target due to its interaction with Hypoxia-inducible factor 1α (HIF1α), which is closely related to the proliferation, survival, self-renewal, and invasiveness of glioma stem cells [59]. The interaction of RUNX1T1 with HIF1α results in a cascade response. After the recruitment of the HIF1α modification factors PHD2 and GSK3β, HIF1α hydroxylation is initiated along with the gathering of E3 ligase, which degrades HIF1α through ubiquitination [60]. Thus, the progression and prognosis of gliomas probably correlate with RUNX1T1 as a core regulator in glioblastoma cell proliferation. In ovarian cancer, RUNX1T1, which is believed to inhibit the proliferation, migration, and invasion of ovarian cancer cells, is promoted when the long non-coding RNA (IncRNA) *EPB41L4A-AS2* is overexpressed. IncRNA sequesters functions by binding with miR-103a to increase RUNX1T1 expression, highlighting the effects of RUNX1T1 and effectively delays ovarian cancer progression [61].

Even though RUNX1T1 is downregulated in most cases of carcinogenesis, its upregulation has also been revealed in research on small cell lung cancer (SCLC) and bladder cancer [14, 52]. By binding to the promoter of CDKN1A, RUNX1T1 inhibits histone acetylation, which leads to E2F upregulation and carcinogenesis acceleration, an expected modulation accompanying the loss of tumor-repressor RB1 in SCLC [14]. RUNX1T1 is also involved in the progression of bladder cancer via the positive RUNX1T1/TCF4/miR-625-5p feedback loop, closely relating to RNA-binding motif protein 24 (RBM24), a determinant of carcinogenesis. The enhancement of *RUNX1T1* mRNA causes a cascade reaction of the loop, causing RBM24 upregulation, further promoting bladder cancer progression [52, 53]. Despite the abundant evidence that RUNX1T1 is closely related to carcinogenesis, it is unknown whether common rules exist between the occurrence of solid tumors and RUNX1T1 expression.

## Conclusion

Although RUNX1T1 has long been identified for its role in the hematopoietic system, recent advances have confirmed its extensive activity in many other cellular functions. However, apart from its main role as a transcription repressor, the effects of RUNX1T1 on cells from different origins and sites can be completely different, largely due to its role as the point of intersection in a complicated crosstalk network between cellular signaling pathways. Thus, there remain numerous questions about the inherent mechanisms of RUNX1T1 on cellular development. Fortunately, using modern technologies, including siRNAs and genome editing technologies, investigations into the control of RUNX1T1 and other target genes have become accessible and greatly facilitated. Further research into accurate mechanisms by which RUNX1T1 functions across different stages and regions may enable RUNX1T1 to become a potential therapeutic target or biomarker, offering remarkable benefits to future clinical treatments.

## Data Availability

Not applicable.

## Abbreviations

AML: Acute myeloid leukemia
BCL6: B-cell lymphoma-6
C/EBP: CCAAT enhancer-binding protein
CACs: Circulating angiogenetic cells
CDK2: Cyclin-dependent kinases 2
ChIP: Chromatin immunoprecipitation
COX2: Cyclooxygenase-2
CRC: Colorectal cancer
CRD: Core repressor domain
DBZ: Dibenzazepine
Dll: Delta-like
DNMT1: DNA methyltransferase-1
ECFCs: Endothelial colony-forming cells
ECs: Endothelial cells
EPCs: Endothelial progenitor cells
ERα: Estrogen receptor α
FGF: Fibroblast growth factors
FOXO1: Forkhead box protein 01
FTO: Fat mass and obesity
GFI1: Growth factor independence-1
HDACs: Histone deacetylases
HHR: Hydrophobic heptad repeat
HIF1α: Hypoxia-inducible factor 1α
HPCs: Hematopoietic progenitor cells
IncRNA: Long non-coding RNA
LAT2: L-aminoacid transporter-2
MTG: Myeloid translocation gene
MYND: Myeloid-Nervy-DEAF1 homology domain
N-CoR: Nuclear core repressor
NHR: Nervy homology region
PETs: Pancreatic endocrine tumors
PLZF: Promyelocytic leukemia zinc finger protein
PPARγ: Peroxisome proliferator-activated receptor gamma
pRb: Phosphorylates retinoblastoma protein
RBM24: RNA-binding motif protein 24
RGCs: Radial glial cells
RUNX1T1: Runt-related transcription factor 1, translocated to 1
SAHA: Suberoylanilide hydroxamic acid
SCLC: Small cell lung cancer
SHARP: SMRT- and HDAC1-associated repressor protein
siRNAs: Small-interfereing RNAs
TMX: Tamoxifen
TFF1: Trefoil factor 1
VEGF: Vascular endothelial growth factor
